# Trends, Perioperative Adverse Events, and Survival of Patients With Left Ventricular Assist Devices Undergoing Noncardiac Surgery

**DOI:** 10.1001/jamanetworkopen.2020.25118

**Published:** 2020-11-12

**Authors:** Amgad Mentias, Alexandros Briasoulis, Mary S. Vaughan Sarrazin, Paulino A. Alvarez

**Affiliations:** 1 Division of Cardiovascular Diseases, Department of Internal Medicine, University of Iowa Hospitals and Clinics, Iowa City; 2 Heart and Vascular Institute, Cleveland Clinic, Cleveland, Ohio; 3 Institute for Clinical and Translational Sciences, The University of Iowa, Iowa City

## Abstract

**Importance:**

Information regarding the performance and outcomes of noncardiac surgery (NCS) in patients with left ventricular assist devices (LVADs) is scarce, with limited longitudinal follow-up data that are mostly limited to single-center reports.

**Objective:**

To examine the trends, patient characteristics, and outcomes associated with NCS among patients with LVAD.

**Design, Setting, and Participants:**

This cohort study examined patients enrolled in Medicare undergoing durable LVAD implantation from January 2012 to November 2017 with follow-up through December 2017. The study included all Medicare Provider and Analysis Review Part A files for the years 2012 to 2017. Patients identified by *International Classification of Diseases, Ninth Revision Clinical Modification *(*ICD-9-CM*) and *International Classification of Diseases, Tenth Revision *(*ICD-10*) procedure codes for new LVAD implantation were included. Data analysis was performed from November 2019 to February 2020.

**Exposures:**

NCS procedures were identified using the *ICD-9-CM* and *ICD-10* procedural codes and divided into elective and urgent or emergent.

**Main Outcomes and Measures:**

The primary outcome was major adverse cardiovascular events (MACEs), defined as in-hospital or 30-day all-cause mortality, ischemic stroke, or intracerebral hemorrhage after NCS. Early (<60 days after NCS) and late (≥60 days after NCS) mortality after NCS were analyzed in both subgroups using time-varying covariate and landmark analysis using patients who did not undergo NCS as reference.

**Results:**

Of the 8118 patients with LVAD (mean [SD] age, 63.4 [10.8] years; 6484 men [79.9%]), 1326 (16.3%, or approximately 1 in 6) underwent NCS, of which 1000 procedures (75.4%) were emergent or urgent and 326 (24.6%) were elective. There was no difference in age between patients who underwent NCS and patients who did not (mean [SD] age, 63.6 [10.6] vs 63.4 [10.9] years). The number of NCS procedures among patients with LVAD increased from 64 in 2012 to 304 in 2017. The median (interquartile range) time from LVAD implantation to NCS was 309 (133-606) days. The most frequent type of NCS was general (613 abdominal, pelvic, and gastrointestinal procedures [46.2%]). Perioperative MACEs occurred in 169 patients (16.9%) undergoing emergent or urgent NCS and 23 patients (7.1%) undergoing elective NCS. Urgent or emergent NCS was associated with higher mortality early (adjusted hazard ratio [aHR], 8.78; 95% CI, 7.20-10.72; *P* < .001) and late (aHR, 1.71; 95% CI, 1.53-1.90; *P* < .001) after NCS compared with patients with LVAD who did not undergo NCS. Elective NCS was also associated with higher mortality early (aHR, 2.65; 95% CI, 1.74-4.03; *P* < .001) and late (aHR, 1.29; 95% CI, 1.07-1.56; *P* = .008) after NCS.

**Conclusions and Relevance:**

One of 6 patients with LVAD underwent NCS. Perioperative MACEs were frequent. Higher mortality risk transcended the early postoperative period in urgent or emergent and elective surgical procedures.

## Key Points

**Question:**

What are the trends, perioperative adverse events, and mortality associated with noncardiac surgery in patients with left ventricular assist devices (LVADs)?

**Findings:**

In this cohort study, of 8118 Medicare patients who had an LVAD, 16.3% underwent noncardiac surgery during follow-up. Major adverse cardiovascular events, defined as in-hospital or 30-day all-cause mortality, ischemic stroke, or intracerebral bleeding, occurred in 16.9% of patients who underwent emergent or urgent procedures and in 7.1% of patients who underwent elective procedures.

**Meaning:**

A substantial proportion of patients with LVADs underwent noncardiac surgery and experienced major perioperative adverse events.

## Teaser

This cohort study examines the trends, patient characteristics, and outcomes associated with noncardiac surgery among patients with left ventricular assist devices (LVADs).

## Introduction

Left ventricular assist devices (LVADs) as a bridge to transplant, a bridge to recovery, or a destination therapy have become standard of care for selected patients with advanced heart failure (HF).^1,2^ Their use has steadily increased, and since 2012 more than 2000 implants have been performed each year in the US.^3^ LVAD therapy has evolved through improvements in device technology and the performance of pragmatic clinical trials.^4,5,6^ One-year survival increased from 52% in 2009 to 83% in 2018.^6,7^ The current mean duration of support is 20 months, and reports of patients who have had an LVAD for 4 years or more are increasing.^3,8,9^ Nevertheless, morbidity remains high with estimated 1-year readmission rates of 80%, gastrointestinal bleeding rates of 25%, and stroke rates of 13%.^3,10^

Approximately 50 million noncardiac surgery (NCS) procedures are performed every year in the US.^11^ It has been estimated that 1.4% to 3.0% of patients will experience a perioperative cardiac event (30-day death, myocardial infarction, or cardiac arrest).^11^ The presence of HF increases the risk of 30-day postoperative mortality. HF is a key component of surgical risk stratification indices.^11,12,13^ The performance of NCS in patients with HF supported with LVADs has been described since their inception in clinical practice, and single-center reports have been increasing.^14,15,16,17,18,19,20,21,22,23,24,25^ However, contemporary national data regarding NCS in patients with LVAD are scarce.The purpose of our study was to evaluate national trends, outcomes, and risk factors associated with complications of NCS in patients with LVAD using a large administrative database of hospital admissions.

## Methods

### Study Cohort

The institutional review board of the University of Iowa approved the study with a waiver for individual informed consent because the study was retrospective and posed minimal risk to the participants. This study followed the Strengthening the Reporting of Observational Studies in Epidemiology (STROBE) reporting guideline.

Medicare patients who underwent new LVAD placement from January 2012 to November 2017 were identified from the 100% Medicare Provider and Analysis Review Part A files. These files included all general hospital admissions for Medicare beneficiaries during a given year and were obtained from the Centers for Medicare & Medicaid Services. LVAD placement was identified using *International Classification of Diseases, Ninth Revision, Clinical Modification *(*ICD-9-CM*) and *International Classification of Diseases, Tenth Revision *(*ICD-10*) procedure codes (02HA0QZ and 3766), with *ICD-9-CM* codes used for discharges through September 2015 and* ICD-10* codes used for the period after September 2015. Patient characteristics, including age, sex, and race, were extracted from Medicare Beneficiary Summary files, and comorbidities were derived from Medicare inpatient claims during the 1 year before the LVAD placement admission. A unique patient identifier was used to link all admissions for each patient. We excluded patients who were enrolled in Medicare for less than 1 year before the LVAD placement date. We used comorbidity algorithms originally defined by Elixhauser et al.^26^

Patients were followed until December 2017, and patients who underwent NCS during follow-up after discharge from LVAD placement admission were identified. We included the following NCS categories: general (included abdominal, pelvic, and gastrointestinal procedures), thoracic, genitourinary, vascular, orthopedic, and head and neck procedures. We excluded 673 patients who underwent neurological procedures to avoid confounding by surgical procedures done for the management of stroke or cerebral hemorrhage. Eleven breast and 27 gynecological procedures were excluded because of their low prevalence. Eighty-three patients who underwent tracheostomy and 92 patients who underwent gastrostomy were excluded because those procedures are usually performed to support critically ill patients. Seventeen procedures with missing surgery dates were excluded. We also excluded patients who died during LVAD placement surgery. If a patient underwent more than 1 surgery during follow-up, the first surgery was used in the study. Study cohort flowchart is shown in the eFigure in the Supplement. We analyzed outcomes of elective surgery separate from urgent or emergent surgical procedures. The urgency of surgery was determined through the inpatient admission type code in Medicare claims.

### Outcomes

The primary outcome of our study was perioperative major cardiovascular adverse events (MACEs), defined as a composite point of in-hospital or 30-day all-cause mortality, ischemic stroke, or intracerebral hemorrhage after NCS. We analyzed early (<60 days after NCS) and late (≥60 days after NCS) mortality after NCS in both subgroups. When assessing in-hospital outcomes, to ensure that an outcome was a new event and not a previous diagnosis, we used the present at admission indicator in the inpatient admission claims.^27^ Secondary end points included individual outcomes of the composite end point, in addition to acute kidney injury (AKI), acute HF, sepsis, blood transfusion, and length of hospital stay. *ICD-9-CM* and* ICD-10* diagnosis codes used to define secondary outcomes are reported in eTable 1 in the Supplement.

### Statistical Analysis

First, we compared characteristics of patients with LVAD who did and did not undergo NCS during follow-up. We used analysis of variance or Wilcoxon test as appropriate for continuous variables and χ^2^ tests for categorical variables. All tests were 2-sided, and significance was set at *P* < .05. For patients who underwent NCS, to determine important factors associated with the composite end point of in-hospital or 30-day MACE, we performed a multivariable logistic regression and calculated adjusted odds ratios (aORs) and 95% CIs. In that model, we updated the age and comorbidities of patients who underwent NCS to reflect their status at the time of NCS admission. The model was adjusted for age, sex, comorbidities, and type of surgery, and then a stepwise backward selection process was performed by dropping variables with *P* > .10, one variable at a time, and also guided by lowest Akaike information criterion with insight from clinical experience and previous published data. Because of the limitations of standard Kaplan-Meier testing to account for the time-dependent variable of NCS,^28^ we performed the Mantel-Byar test to compare all-cause mortality between patients with LVAD who did and did not undergo NCS.^29^ The results of the tests were plotted using the nonparametric Simon-Makuch plot, which accounts for the time dependency of NCS.^30^ Because the hazard of mortality after NCS was not uniform across time, we used a time-varying covariant in the Cox regression model to estimate the adjusted hazard ratio (aHR) of mortality early (<60 days) and later (≥60 days) after NCS.

All analysis was performed with SAS statistical software version 9.4 (SAS Institute) and R statistical software version 3.4.3 (R Project for Statistical Computing). Data analysis was performed from November 2019 to February 2020.

## Results

Of the 8118 patients with LVAD included in the study (mean [SD] age, 63.4 [10.8] years; 6484 men [79.9%]), 1326 (16.3%, or approximately 1 in 6 patients) underwent NCS (eFigure in the Supplement). There was no difference in age between patients who underwent NCS and patients who did not (mean [SD], 63.6 [10.6] vs 63.4 [10.9] years). Patients who underwent NCS were more likely than patients who did not undergo NCS to be male (1104 patients [83.3%] vs 5380 patients [79.2%]) and to have a history of revascularization, smoking, and pulmonary hypertension (Table 1). The number of NCS procedures performed for patients with LVAD increased from 64 in 2012 to 304 in 2017. Of the admissions, 1000 (75.4%) were for emergent or urgent NCS procedures and 326 (24.6%) were for elective NCS procedures. The median (interquartile range) time from LVAD implantation to NCS was 309 (133-606) days. The most frequent class of NCS was general (613 procedures [46.2%]) followed by thoracic (219 procedures [16.5%]), orthopedic (199 procedures [15.0%]), genitourinary (154 procedures [11.6%]), head and neck (40 procedures [3.0%]), and vascular (101 procedures [7.6%]) procedures. The types of elective and emergent or urgent surgical procedures are shown in Table 2.

**Table 1.** Baseline Characteristics of the Study Cohort

Variable	Patients with LVAD, No. (%)		
	Overall (N = 8118)	Without NCS (n = 6792)	With NCS (n = 1326)
Age, mean (SD), y	63.4 (10.8)	63.4 (10.9)	63.6 (10.6)
Male	6484 (79.9)	5380 (79.2)	1104 (83.3)
Race/ethnicity			
White	5672 (69.9)	4723 (69.5)	949 (71.6)
Black	1908 (23.5)	1611 (23.7)	297 (22.4)
Hispanic	191 (2.4)	159 (2.3)	32 (2.4)
Alcohol use disorder	202 (2.5)	179 (2.6)	23 (1.7)
Anemia	2264 (27.9)	1912 (28.2)	352 (26.6)
Connective tissue disease	218 (2.7)	181 (2.7)	37 (2.8)
Lung disease	2490 (30.7)	2078 (30.6)	412 (31.1)
Coagulopathy	1082 (13.3)	902 (13.3)	180 (13.6)
Depression	1438 (17.7)	1204 (17.7)	234 (17.7)
Diabetes	3602 (44.4)	2988 (44.0)	614 (46.3)
Drug use disorder	202 (2.5)	172 (2.5)	30 (2.3)
Hypertension	6044 (74.5)	5080 (74.8)	964 (72.7)
Hypothyroidism	1359 (16.7)	1132 (16.7)	227 (17.1)
Liver disease	445 (5.5)	373 (5.5)	72 (5.4)
Lymphoma	99 (1.2)	81 (1.2)	18 (1.4)
Electrolytes abnormalities	4208 (51.8)	3515 (51.8)	693 (52.3)
Obesity	2099 (25.9)	1743 (25.7)	356 (26.9)
Peripheral arterial disease	1078 (13.3)	877 (12.9)	201 (15.2)
Psychosis or dementia	245 (3.0)	217 (3.2)	28 (2.1)
Pulmonary hypertension	2166 (26.7)	1736 (25.6)	430 (32.4)
Tumor	185 (2.3)	158 (2.3)	27 (2.0)
Weight loss	679 (8.4)	563 (8.3)	116 (8.8)
Prior cerebral hemorrhage	0	23 (0.4)	0
Coronary artery disease	5348 (65.9)	4459 (65.7)	889 (67.0)
Smoking	2586 (31.9)	2107 (31.0)	479 (36.1)
Prior revascularization	2771 (34.1)	2254 (33.2)	517 (39.0)
Prior ischemic stroke	334 (4.1)	270 (4.0)	64 (4.8)
Implantable defibrillator	5655 (69.7)	4745 (69.9)	910 (68.6)
Pacemaker	1013 (12.5)	843 (12.4)	170 (12.8)
Chronic kidney disease	4051 (49.9)	3408 (50.2)	643 (48.5)
End stage renal disease	93 (1.2)	73 (1.1)	20 (1.5)
Prior bleeding	893 (11.0)	756 (11.1)	137 (10.3)
Preexisting atrial fibrillation	4812 (59.3)	4063 (59.8)	749 (56.5)

Abbreviations: LVAD, left ventricular assist device; NCS, noncardiac surgery.

**Table 2.** Type of Noncardiac Surgery in Patients With Left Ventricular Assist Devices

Surgery category	Procedures, No.		
	Overall	Urgent	Elective
General		458	155
Hernia repair	142	89	53
Cholecystectomy	107	80	27
Colorectal resection	65	48	17
Gastrectomy	63	36	27
Colostomy or ileostomy	62	NA^a^	NA^a^
Peritoneal adhesiolysis	55	40	15
Other lower gastrointestinal tract surgery	49	NA^a^	NA^a^
Appendectomy	26	NA^a^	NA^a^
Exploratory laparotomy or splenectomy	23	NA^a^	NA^a^
Small bowel resection	21	NA^a^	NA^a^
Thoracic		194	25
Other thoracic surgery	206	NA^a^	NA^a^
Lobectomy	13	NA^a^	NA^a^
Orthopedic		137	62
Excision of bone	71	NA^a^	NA^a^
Lower extremity amputation	58	43	15
Hip or knee replacement	57	30	27
Laminectomy or spinal fusion	13	NA^a^	NA^a^
Genitourinary		106	48
Removal of urinary obstruction or other urinary procedures	71	52	19
Nephrostomy or nephrectomy	50	35	15
Transurethral resection of the prostate or prostatectomy	33	19	14
Head and neck		29	11
Ear, nose, mouth, and pharynx	27	NA^a^	NA^a^
Thyroidectomy	13	NA^a^	NA^a^
Vascular		75	26
Endarterectomy or aortic resection	67	56	11
Peripheral bypass	34	19	15

Abbreviation: NA, not available.

^a^ Cells for which the number is less than 11 are suppressed per Center for Medicare and Medicaid Services policy.

**Figure 1.**
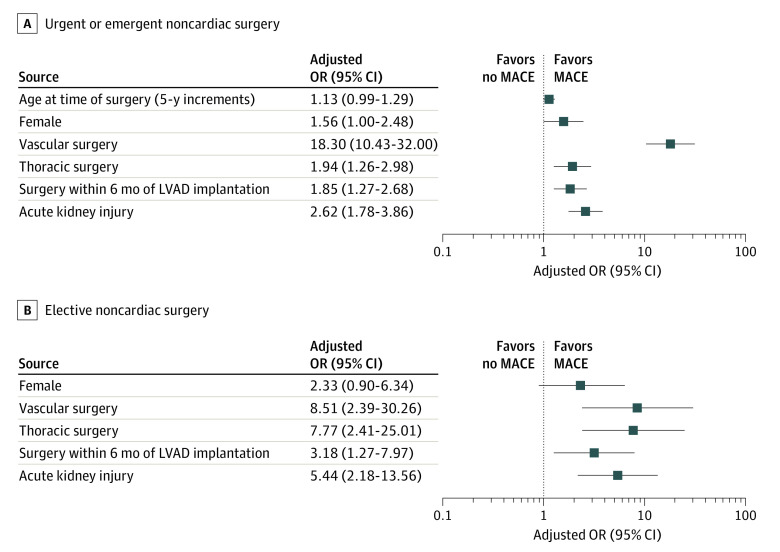
Factors Associated With Major Adverse Cardiovascular Events (MACEs) Among Patients With Left Ventricular Assist Devices (LVADs) Who Underwent Emergent or Urgent and Elective Noncardiac Surgery Adjusted odds ratios (ORs) and 95% CIs are shown for patients who underwent emergent or urgent (A) and elective (B) noncardiac surgery.

**Figure 2.**
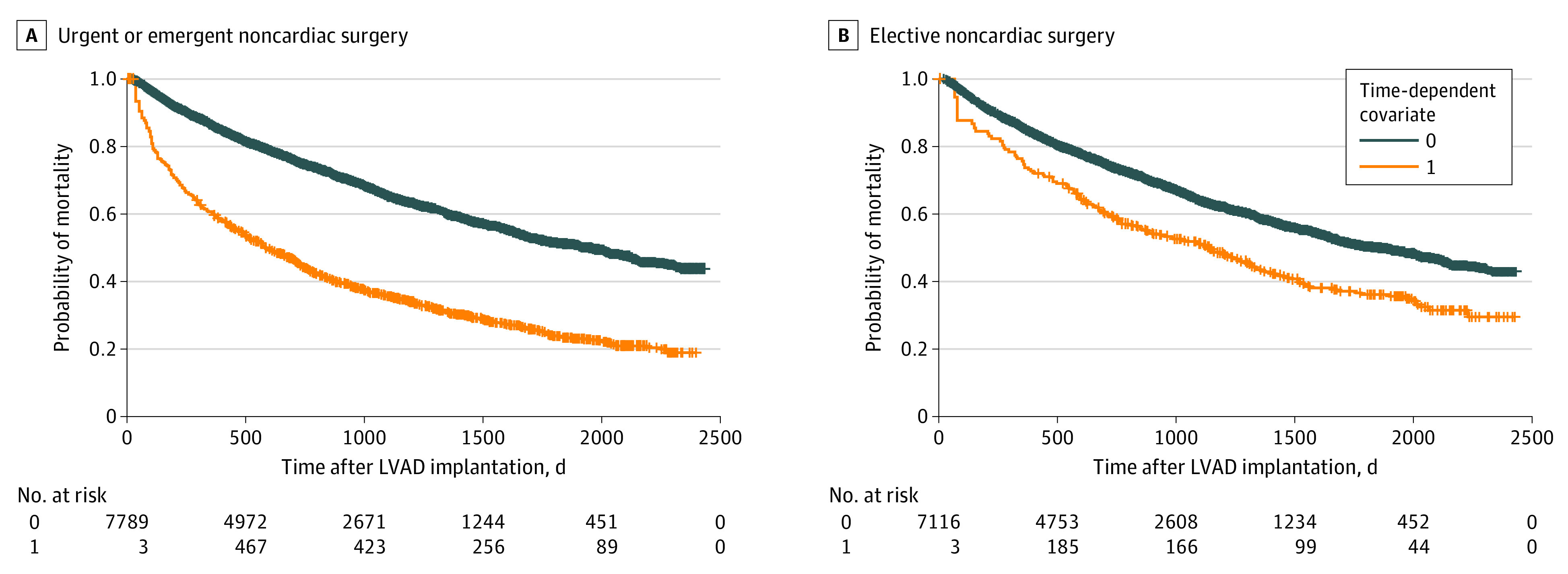
Simon-Makuch Curves for All-Cause Mortality Among Patients With Left Ventricular Assist Devices (LVADs) After Emergent or Urgent and Elective Noncardiac Surgery Graphs show probability of mortality for patients with LVAD after emergent or urgent (A) and elective (B) noncardiac surgery who did not have noncardiac surgery used as a reference (*P* < .01, Mantel-Byar test, for both comparisons).

Adverse events, length of stay, and 30-day readmission rates according to surgery urgency are shown in Table 3. Perioperative MACEs occurred in 169 patients (16.9%) undergoing emergent or urgent NCS and 23 patients (7.1%) undergoing elective NCS. A total of 116 patients (8.7%) died within 30 days. Of the patients who underwent NCS, 493 (37.2%) were readmitted within 30 days, and 453 (34.2%) received blood transfusion. After multivariable adjustment, factors associated with perioperative MACE in urgent or emergent and elective NCS were vascular surgery (aOR, 18.30 [95% CI, 10.43-32.00] for urgent or emergent NCS and 8.51 [95% CI, 2.39-30.26] for elective NCS), thoracic surgery (aOR, 1.94 [95% CI, 1.26-2.98] for urgent or emergent NCS and 7.77 [95% CI, 2.41-25.01] for elective NCS), surgery within 6 months of LVAD implantation (aOR, 1.85 [95% CI, 1.27-2.68] for urgent or emergent NCS and 3.18 [95% CI, 1.27-7.97] for elective NCS), and postoperative AKI (aOR, 2.62 [95% CI, 1.78-3.86] for urgent or emergent NCS and 5.44 [95% CI, 2.18-13.56] for elective NCS) (Figure 1). Urgent or emergent NCS was associated with higher mortality in the early postoperative period (<60 days after NCS; aHR, 8.78; 95% CI, 7.20- 10.72; *P* < .001) and also later in follow-up (≥60 days after NCS; aHR, 1.71; 95% CI, 1.53-1.90; *P* < .001) compared with patients with LVAD who did not undergo surgery (Figure 2A and eTable 2 in the Supplement). Elective NCS was also associated with higher mortality in the early postoperative period (aHR, 2.65; 95% CI, 1.74-4.03; *P* < .001) and later during follow-up (aHR, 1.29; 95% CI, 1.07-1.56; *P* = .008) (Figure 2B and eTable 3 in the Supplement).

**Table 3.** Adverse Perioperative Outcomes According to Type of Surgery

Variable	Patients, No. (%)		*P* value
	Emergent or urgent surgery (n = 1000)	Elective surgery (n = 326)	
30-d Mortality	102 (10.2)	14 (4.3)	.001
In-hospital or 30-d			
Ischemic stroke	68 (6.8)	NA^a^	.003
Brain bleed	33 (3.3)	NA^a^	.009
Acute kidney injury	455 (45.5)	82 (25.2)	<.001
Sepsis	103 (10.3)	18 (5.5)	.009
Acute heart failure	101 (10.1)	27 (8.3)	.30
Blood transfusion	375 (37.5)	78 (23.9)	<.001
Length of stay, median (interquartile range), d	13 (8-23)	8 (5-13)	<.001
30-d Any readmission	387 (38.7)	106 (32.5)	.045
Major adverse cardiovascular event	169 (16.9)	23 (7.1)	<.001

Abbreviation: NA, not available.

^a^ Cells for which the number is less than 11 are suppressed per Center for Medicare and Medicaid Services policy.

## Discussion

In this analysis of a large administrative database of patients with LVAD who underwent NCS, we report several findings. First, approximately 1 in 6 patients with LVAD underwent NCS after being discharged from the index hospitalization for LVAD implantation. Furthermore, the number of NCS procedures increased over the study years. Second, perioperative MACEs occurred in 7.1% of patients undergoing elective NCS and in 16.9% of patients undergoing urgent or emergent NCS. Third, the type of surgery, timing after LVAD implantation (within 6 months), and development of AKI after NCS were independently associated with MACE. In addition, the risk of mortality in patients who underwent NCS was higher both early (<60 days) and late (≥60 days) after the index NCS. In addition, 493 (37.2%) of the patients who underwent NCS were readmitted within 30 days, and 453 (34.2%) received blood transfusion.

In a previous report,^31^ the perioperative mortality of Medicare patients with HF, but without LVAD, undergoing NCS from 1997 to 1998 was 11.7%, compared with 6.6% in patients with coronary artery disease and 6.2% among controls. In a recent analysis^32^ of the Veterans Health Administration System, 90-day postoperative mortality among patients with a history of HF was 5.5% compared with 1.2% in those without HF. In our study, the overall postoperative mortality rate was 8.7%, which is comparable to the previously reported estimates, in the context of patients with stage D HF and the challenges of LVAD management.

An analysis^33^ of the National Inpatient Sample from 2007 to 2010 reported the outcomes of 298 patients with LVAD who underwent NCS: the inpatient mortality rate of the NCS group was 22.8% and was not significantly different from that for patients who did not undergo NCS (17.9%; *P* = .10). Potential explanations of the higher mortality compared with our study include the fact that we excluded patients who underwent NCS during the index hospitalization for LVAD implantation, and our study included more contemporary generations of LVADs implanted from 2012 to 2017. In single-center reports^16,17,21^ of patients with durable, continuous LVADs undergoing NCS, the perioperative mortality rates ranged from 0% to 16%. This variation in mortality rates appears to be attributable to heterogeneity in the inclusion criteria. For example, in the study by Nelson et al,^21^ almost one-half of the procedures were endoscopies performed for gastrointestinal bleeding. Elective NCS constituted 88% of the procedures in the report by Morgan et al.^16^ Bhat et al,^17^ who reported the highest perioperative mortality rate, included neurosurgical procedures, and 50% of the deaths in that study occurred after neurosurgical procedures. We excluded endoscopies, given that they are considered a minor procedure. We also excluded neurosurgical procedures because one of our end points was stroke, and it would be difficult to differentiate whether a stroke was a reason for vs a result of a neurosurgical procedure.^34^ Intrathoracic and vascular surgical procedures are known to be associated with higher risk of cardiac events and mortality and are included in most preoperative risk stratification measures.^35^ Our results confirm that this is also true in patients with HF supported with LVAD.

In our study, postoperative AKI and undergoing NCS within 6 months of LVAD implantation were significantly associated with worse outcomes. Thus, it is probably reasonable to postpone NCS, if possible, for few months after a new LVAD is implanted. Furthermore, it is important to optimize fluid status of these patients in the perioperative period, to reduce the risk of postoperative AKI as much as possible. The experience in performing NCS in patients with LVAD has been accumulating, reflected by the increase in both original research and systematic and narrative reviews of the subject.^36,37,38,39,40,41^ In our study, the number of NCS procedures performed for patients with LVAD increased over the years. Recommendations for monitoring, staffing, and management have been provided in the 2013 International Society of Heart and Lung Transplantation guidelines for mechanical circulatory support.^42^ Most of the recommendations are based on expert opinion, and the heterogeneity in resource utilization for NCS in patients with LVAD has been reported.^43^ Our findings highlight the need for collaborative research in the perioperative management of patients with LVAD to answer important questions, such as management of anticoagulation and antiplatelets in the perioperative period, and the optimum timing of elective NCS after LVAD placement.

### Limitations

To our knowledge, our study represents the largest analysis of NCS in patients with LVAD that reported outcomes for both elective and emergent or urgent procedures and provided long-term follow-up. However, limitations should be recognized. First, the lack of hemodynamic and echocardiographic data precludes evaluation of the presence and severity of right ventricular dysfunction, which has been associated with worse survival in patients with LVAD and could potentially play an important role in risk stratification.^44^ Second, we were not able to define specific LVAD models.^45^ During the study period, only continuous-flow LVADs were implanted in the US.^3^ Third, we were not able to define whether the indication for LVAD was a bridge to transplant or a destination therapy. Fourth, because our study cohort was derived from an administrative database, there is potential for misclassification because of coding errors, especially with the transition from *ICD-9-CM* to *ICD-10 *codes.^46^ Furthermore, approximately three-quarters of the cases were urgent or emergent. This means than an acute process is associated with the need for surgery and may affect the outcomes. This critical consideration should be kept in mind when interpreting the conclusions of our study.

## Conclusions

NCS in patients with LVAD is frequent and associated with high morbidity and mortality. Factors associated with adverse outcomes included the type of surgery, the timing of operation, and the development of AKI. The proportion of patients who are readmitted and who receive red blood cell transfusion is high even for elective procedures. Further prospective studies to improve risk stratification and inform perioperative management of NCS in patients with LVAD are warranted.

## Notes

Supplement. **eFigure.** Flow Chart for the Study Cohort **eTable 1.** *ICD-9* and *ICD-10* Codes Used to Define Study Secondary Outcomes **eTable 2.** Full Cox Regression Model for All-Cause Mortality With Urgent Noncardiac Surgery in LVAD Patients **eTable 3.** Full Cox Regression Model for All-Cause Mortality With Elective Noncardiac Surgery in LVAD Patients
