# Supplementary material for: Trends, Perioperative Adverse Events, and Survival of Patients With Left Ventricular Assist Devices Undergoing Noncardiac Surgery
Source: JAMA Netw Open. 2020 Nov 12;3(11):e2025118. doi: 10.1001/jamanetworkopen.2020.25118 (PMC7662145; doi:10.1001/jamanetworkopen.2020.25118)
Supplement: Supplement. — eFigure. Flow Chart for the Study Cohort eTable 1. ICD-9 and ICD-10 Codes Used to Define Study Secondary Outcomes eTable 2. Full Cox Regression Model for All-Cause Mortality With Urgent Noncardiac Surgery in LVAD Patients eTable 3. Full Cox Regression Model for All-Cause Mortality With Elective Noncardiac Surgery in LVAD Patients [file jamanetwopen-e2025118-s001.pdf]

Supplemental Online Content

Mentias A, Briasoulis A, Vaughan Sarrazin MS, Alvarez PA. Trends, perioperative adverse events, and survival of patients with left ventricular assist devices undergoing noncardiac surgery. *JAMA Netw Open*. 2020;3(11):e2025118. doi:10.1001/jamanetworkopen.2020.25118

eFigure. Flow Chart for the Study Cohort

eTable 1. *ICD-9* and *ICD-10* Codes Used to Define Study Secondary Outcomes

eTable 2. Full Cox Regression Model for All-Cause Mortality With Urgent Noncardiac Surgery in LVAD Patients

eTable 3. Full Cox Regression Model for All-Cause Mortality With Elective Noncardiac Surgery in LVAD Patients

This supplemental material has been provided by the authors to give readers additional information about their work.

© 2020 Mentias A et al. *JAMA Network Open.*

eFigure: Flow Chart for the Study Cohort

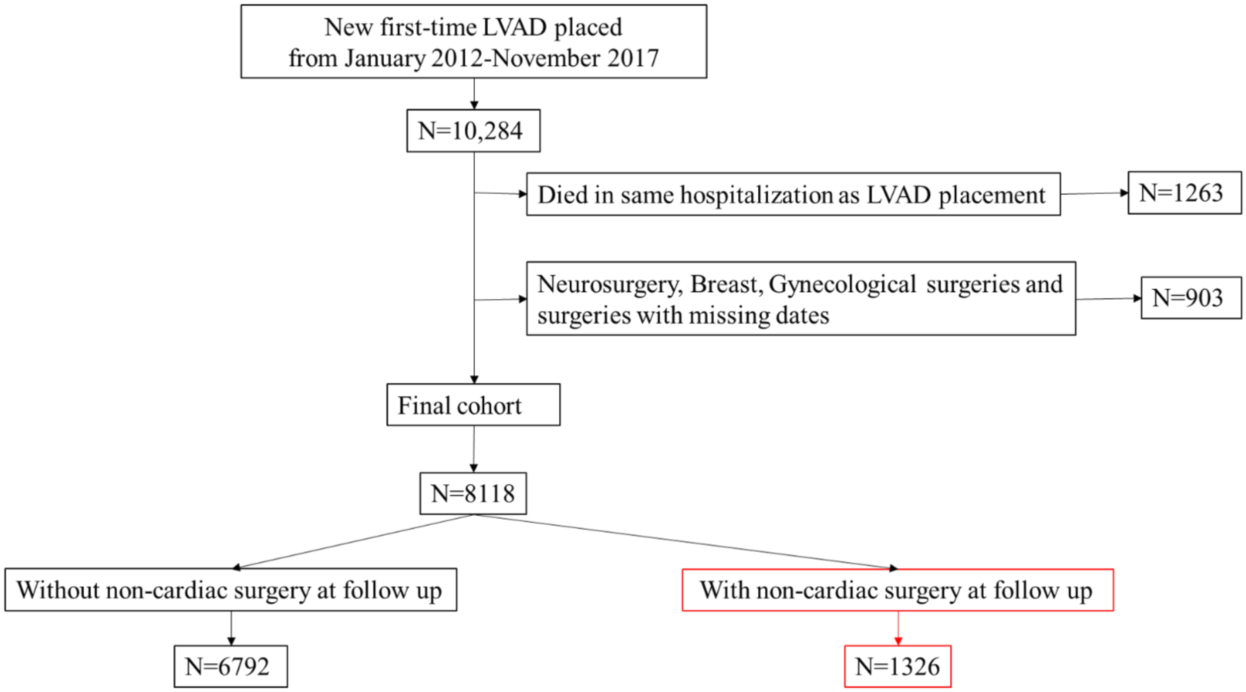

© 2020 Mentias A et al. *JAMA Network Open.*

eTable 1. *ICD-9* and *ICD-10* Codes Used to Define Study Secondary Outcomes

Outcome	ICD-9 codes	ICD-10 codes
Acute kidney injury	584.*, 794.4	N17.*, N99.0, R94.4
Ischemic Stroke	433, 434, 436	I63.*, I65.*, I66.*
Cerebral hemorrhage	430, 431, 432	I60.*, I61.*, I62.*
Acute Heart failure	'42821','42823' ,'42831','42833','42841','42843'	I50.21, I50.23, I50.31, I50.33, I50.41, I50.43
Sepsis	995.91, 995.92, 998.02, 785.52, 422.92, 038.*	A41.*, R65.2, T81.44, T81.12, A40.*

© 2020 Mentias A et al. *JAMA Network Open.*

eTable 2. Full Cox Regression Model for All-Cause Mortality With Urgent Noncardiac Surgery in LVAD Patients

Variable	HR	95% CI	P value
Late urgent surgery	1.71	1.53-1.91	<0.001
Early urgent surgery	8.78	7.21-10.72	<0.001
Age (per 1-year increment)	1.01	1.01-1.02	<0.001
Black race	0.87	0.79-0.96	0.006
Female sex	1.11	1.01-1.22	0.03
Chronic lung disease	1.25	1.16-1.36	<0.001
Diabetes	1.18	1.11-1.27	<0.001
Lymphoma	1.53	1.16-2.02	0.003
Metastasis	2.42	1.37-4.29	0.002
Prior coronary revascularization	1.19	1.1-1.29	<0.001
Prior ICD	0.85	0.78-0.92	<0.001
Chronic kidney disease	1.13	1.05-1.22	0.001
Preexisting atrial fibrillation	1.12	1.04-1.21	0.003

© 2020 Mentias A et al. *JAMA Network Open.*

eTable 3. Full Cox Regression Model for All-Cause Mortality With Elective Noncardiac Surgery in LVAD Patients

Variable	HR	95% CI	P value
Late elective surgery	1.29	1.07-1.56	0.008
Early elective surgery	2.65	1.74-4.03	<0.001
Age (per 1-year increment)	1.01	1.01-1.02	<0.001
Black Race	0.84	0.76-0.93	0.001
Other race	0.85	0.72-1.01	0.06
Female sex	1.10	0.99-1.21	0.07
Connective tissue disease	1.34	1.07-1.67	0.01
Chronic lung disease	1.22	1.12-1.33	<0.001
Diabetes	1.18	1.09-1.28	<0.001
Liver disease	1.20	1.02-1.41	0.03
Lymphoma	1.39	1.02-1.9	0.04
Metastasis	2.14	1.15-3.99	0.02
Prior coronary revascularization	1.24	1.14-1.35	<0.001
Prior ICD	0.83	0.76-0.91	<0.001
Chronic kidney disease	1.11	1.03-1.21	0.008
Preexisting atrial fibrillation	1.13	1.04-1.22	0.004

© 2020 Mentias A et al. *JAMA Network Open.*
